# Draft Genome Sequences of the Oomycete *Pilasporangium apinafurcum* Strains JCM 30513 and JCM 30514, Formerly Classified as *Pythium apinafurcum*

**DOI:** 10.1128/genomeA.00899-17

**Published:** 2017-08-31

**Authors:** Shihomi Uzuhashi, Rikiya Endoh, Ri-ichiroh Manabe, Moriya Ohkuma

**Affiliations:** a Genetic Resources Center, National Agriculture and Food Research Organization, Tsukuba, Ibaraki, Japan; b Microbe Division/Japan Collection of Microorganisms (JCM), RIKEN BioResource Center, Tsukuba, Ibaraki, Japan; c Division of Genomic Technologies, RIKEN Center for Life Science Technologies, Suehiro-cho, Tsurumi-ku, Yokohama, Kanagawa, Japan

## Abstract

*Pilasporangium apinafurcum*, formerly classified as *Pythium apinafurcum*, is a unique oomycete that infects plants asymptomatically. Here, we present the draft genome sequences of two variants of *P. apinafurcum*, JCM 30513 and JCM 30514, isolated from uncultivated field soil in Wakayama Prefecture, Japan.

## GENOME ANNOUNCEMENT

*Pilasporangium apinafurcum* is an oomycete formerly classified as *Pythium apinafucrum* based on its morphological similarity with many *Pythium* spp. (1, 2). The species was transferred from *Pythium* to a new genus, *Pilasporangium*, because of its unique phylogenetic position, and since then, only the single species has been assigned to the genus (3). *P. apinafurcum* infects some plants asymptomatically (1). Two phylogenetic subgroups in *P. apinafurcum* are known. They were distinguished from each other based on the nucleotide sequences of the internal transcribed spacer regions of the rRNA gene and mitochondrial cytochrome *c* oxidase genes 1 and 2, but no apparent difference was observed in terms of morphology or pathogenicity between the two subgroups (1).

Here, we present the draft genome sequences of *P. apinafurcum* strains JCM 30513 and JCM 30514, each belonging to a different subgroup. Methods for cultivation, genomic DNA extraction, and purification were described previously (4). Briefly, *P. apinafurcum* was cultured on potato dextrose agar, and DNA was extracted using phenol-chloroform-isoamyl alcohol followed by purification using the Genomic-tip 100/G (Qiagen) and PowerClean Pro DNA cleanup kit (Mo Bio Laboratories, Inc.). A paired-end (PE) shotgun library and a mate-pair (MP) library were constructed using a TruSeq DNA library prep kit (Illumina) and a Nextera mate-pair library prep kit (Illumina), respectively. Sequencing for the PE and MP libraries was performed using the Illumia HiSeq 2500 platform. The MP reads were processed with NextClip version 0.8 (5) to remove adapter sequences. The PE and MP reads were assembled using ALLPATHS-LG version 52488 (6) into scaffolds with total lengths of 37,582,498 bp (JCM 30513) and 37,444,745 bp (JCM 30514), and the genome sizes and proportions of repetitive regions were 53.05 Mb and 37% and 49.48 Mb and 32%, respectively. The G+C content, number of contigs, number of scaffolds, and *N*_50_ (scaffold) were 43.5%, 899, 310, and 400.6 kb (JCM 30513) and 43.9%, 855, 280, and 449.6 kb (JCM 30514), respectively. Total sequencing depths (total read yields divided by estimated genome size) were 211× (158× for PE, 53× for MP libraries) for JCM 30513 and 278× (217× for PE, 61× for MP libraries) for JCM 30514. Completeness of the assemblies was estimated by CEGMA (7) to be 95.56% (JCM 30513) and 96.37% (JCM 30514), respectively. Gene prediction was performed using the MAKER annotation pipeline version 2.31.8 (8), including AUGUSTUS version 3.0.3 (9), SNAP version 2013-02-16 (10), and GeneMark-ES suite 4.21 (11), where AUGUSTUS and SNAP were trained on *Pythium ultimum* var.* sporangiiferum* BR650 (12); 13,996 (JCM 30153) and 14,069 (JCM 30514) protein-coding genes were predicted. These numbers are similar with those reported for other pythiaceous species (12, 13). Sma3s version 2013-09-01 (14) with the UniProt-TrEMBL release 2015_11 and UniProt-SwissProt release 2015_11 databases enabled functional annotation of 2,665 and 1,369 genes for JCM 30513 and 2,697 and 1,373 genes for JCM 30514, respectively.

The draft genome sequences reported here will contribute to a more detailed understanding of pythiaceous species, including the mechanisms of asymptomatic infection of plants by *P. apinafurcum*.

### Accession number(s).

This whole-genome shotgun project has been deposited in DDBJ/ENA/GenBank under BioProject no. PRJDB3796 (JCM 30513) and PRJDB3797 (JCM 30514) with GenBank accession no. BCKD01000001 to BCKD01000310 (JCM 30513) and BCKE01000001 to BCKE01000280 (JCM 30514). The versions described here are the first versions.
